# A Case of Delirium Tremens Induced by the Inordinate Use of Tobacco

**Published:** 1847-09

**Authors:** Wm. A. Gordon

**Affiliations:** Harrisburg, Missouri


**Art. IV.—*A Case of Delirium Tremens induced by the inordinate Use of Tobacco.* By Wm. A. Gordon, M.D., of Harrisburg, Mo.**

Last spring, while on a visit to my relations in the southern part of Kentucky, I met with the following case of delirium tremens. The patient, aged 71 years, had been smoking tobacco to great excess for a number of years. At length, a short time before I saw him, he resolved to abandon the use of it altogether. The day on which he formed this resolution he smoked, in quick succession, nine cigars, which was followed by considerable nausea and giddiness for three days. These symptoms then passed off and his health for a short time seemed better than usual; but after this brief interval he fell into a lethargic state from which he was with difficulty aroused. This condition was succeeded by the symptoms of a true delirium tremens. He was wakeful, agitated, talkative, and alarmed at imaginary objects around his bed. His pulse was about 85 a minute, full but soft; countenance dejected with a wild confused look; skin cold and moist; bowels constipated; tongue moist and slightly coated.

I am not able to report the termination of this singular case, as I left the neighborhood soon after I saw the patient, but as having a physiological interest, I will mention two phenomena which were reported to me in connexion with it.

1st. The patient previous to this attack had been hard of hearing. While laboring under it his hearing became excellent.

2d. He had also labored under some difficulty of speech, for a number of years, owing to what seemed a partial paralysis of the tongue. When the derangement of the cerebral system came on, he recovered the use of his tongue and was able to speak distinctly and rapidly.

July 1, 1847.

